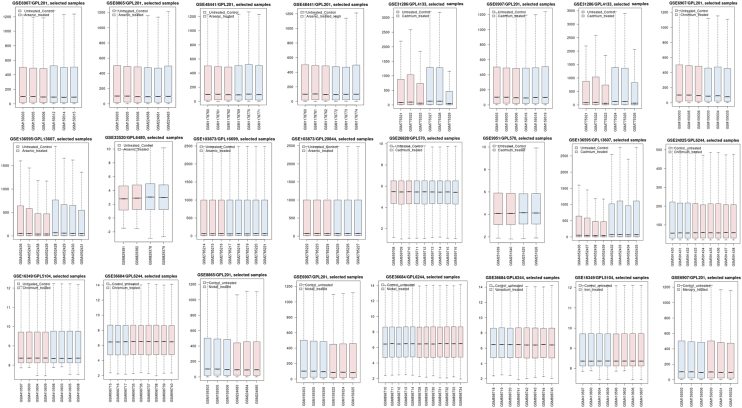# Effects of arsenic and heavy metals on metabolic pathways in cells of human origin: Similarities and differences

**DOI:** 10.1016/j.toxrep.2021.05.015

**Published:** 2021-05-31

**Authors:** Kaniz Fatema, Sabrina Samad Shoily, Tamim Ahsan, Zinia Haidar, Ahmed Faisal Sumit, Abu Ashfaqur Sajib

**Affiliations:** a Department of Genetic Engineering & Biotechnology, University of Dhaka, Dhaka, Bangladesh; b Department of Mathematics and Natural Sciences, Brac University, Dhaka, Bangladesh

**Keywords:** Cell line, Human, Heavy metal, Arsenic, Cadmium, Chromium, Metabolic pathway

## Abstract

Various anthropogenic and natural events over the years have gradually increased human exposure to various heavy metals. Several of these heavy metals including cadmium, mercury, nickel, chromium, and the metalloid arsenic among others, have created major public health concerns for their high level of toxicities. Identification of the general as well as the differentially affected cellular metabolic pathways will help understanding the molecular mechanism of different heavy metal-induced toxicities. In this study, we analyzed 25 paired (control vs. treated) transcriptomic datasets derived following treatment of various human cells with different heavy metals and metalloid (arsenic, cadmium, chromium, iron, mercury, nickel and vanadium) to identify the affected metabolic pathways. The effects of these metals on metabolic pathways depend not only on the metals *per se*, but also on the nature of the treated cells. Tissue of origin, therefore, must be considered while assessing the effects of any particular heavy metal or metalloid. Among the metals and metalloid, arsenic appears to have relatively more pleiotropic influences on cellular metabolic pathways including those known to have association with diabetes. Although only two stem cell derived datasets are included in the current study, effects of heavy metals on these cells appear to be different from other mature cells of similar tissue origin. This study provides useful information about different heavy metal affected pathways, which may be useful in further exploration using wet-lab based techniques.

## Highlights

• There are distinctive overlaps in different heavy metal affected metabolic pathways.
• Affected pathways vary according to the tissue origin and maturity of the cell.
• Arsenic appears to have relatively more pleiotropic effects on metabolic pathways.
• Some of the arsenic affected pathways are associated with diabetes.

## Graphical abstract

**Figure:**
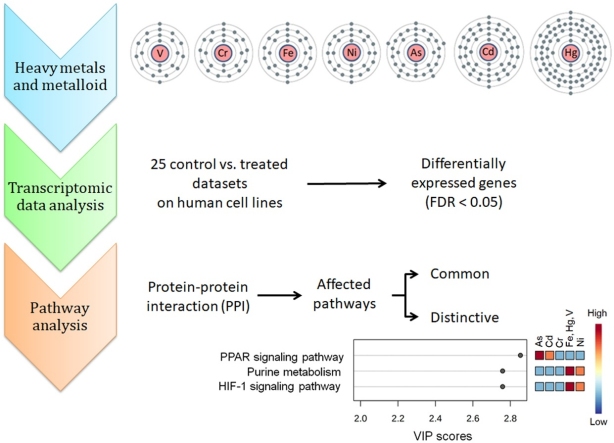

## 1 Introduction

Heavy metals are natural elements characterized by their high atomic weights and specific gravities greater than five [1,2]. Arsenic, which is a metalloid, has a high specific gravity as well. Several of the heavy metals and metalloids, in particular, arsenic, cadmium, chromium, mercury and nickel, among others have created major public health concerns for their high level of toxicities [1,[3], [4], [5], [6]]. Humans are exposed to these heavy metals through anthropogenic activities such as mining and smelting operations together with industrial, agricultural, and domestic use of metals and metal-containing compounds [3,7]. The major sink for the heavy metals released into the environment is soil, where these accumulate over time without undergoing extensive chemical or microbial degradation [8]. These can enter the human body through skin, by inhalation, and through the intake of contaminated food and water [[9], [10], [11]]. Heavy metal mediated toxicity occurs when chronic or acute exposure to a heavy metal exceeds certain threshold levels in the body [12]. Toxicity may result from the interactions of heavy metals with biomolecules like DNA and proteins, and thus modulating important cellular pathways [1]. Reactive oxygen species (ROS) generated due to heavy metal exposure may cause cellular damage in different organs including liver, brain and kidney [1,13].

Arsenic is the 20th most abundant element on earth’s crust [14] and well known as one of the few metals and metalloids that can cause large-scale adverse health effects [12]. Over 140 million people in more than 70 countries around the globe are facing chronic exposure to arsenic through ground water at levels higher than the maximum permissible limit of 10 μg/L set by the World Health Organization (WHO) [15,16]. In Bangladesh, an estimated 35–77 million people have been facing chronic exposure to arsenic through drinking water and food crops- an incident which is dubbed as “the largest mass poisoning of a population in history” [17]. Chronic arsenic toxicity or arsenicosis has previously been associated with skin lesions and cancers of the skin, bladder, lung, kidney, liver, and colon [[18], [19], [20], [21], [22]].

Cadmium (Cd) is another non-essential heavy metal that poses significant health risks to human. Chronic exposure to cadmium leads to systemic toxicity and cancers in the lung, breast, prostate, nasopharynx, pancreas, and kidney [23,24]. The liver and kidneys are extremely sensitive to cadmium toxicity [24,25]. This heavy metal also poses a risk of osteoporosis [24,25]. Exposure to cadmium occurs primarily through the intake of contaminated drinking water, fruits, grains, leafy vegetables and to a considerable extent through cigarette smoking and inhalation [18,24]. More than the maximum permissible limits (0.003 mg/L) of cadmium [12] in drinking water have been reported in countries including Pakistan [26], Nigeria [27], Sweden [28], and Saudi Arabia [29]. Accumulated cadmium in the body typically has a long half-life of about 25–30 years [24]. Although it is a natural element, humans are mostly exposed to cadmium through agricultural and industrial sources [28,30].

Chromium in small amount is an essential element for normal metabolic functions. However, at high concentrations this heavy metal, especially in the hexavalent form (Cr (VI)), it is toxic and carcinogenic [31]. Cr (VI) can form Cr-DNA adduct which can lead to mutation and chromosomal breakage [32]. Chronic inhalation of Cr (VI) affects the respiratory tract [33] and can lead to the development of lung cancer [34] in human. Studies on animal model have shown that long-term chromium exposure can lead to oxidative stress and immunotoxicity [35]. Adverse effects on reproductive organs and reduced fertility was observed as a result of chronic chromium exposure in both male and female mice [36].

Other heavy metals like mercury, nickel, vanadium, etc also have adverse effects on human health. Human exposure to mercury occurs through inhalation of elemental (metallic) mercury vapor via industrial practices, dental amalgam or ingestion of organic mercury compounds (methyl, dimethyl, or ethyl mercury) primarily through consumption of contaminated fish [37]. Mercury affects functions of the renal, endocrine, immune and peripheral nerve systems [37,38]. Children are more susceptible and vulnerable to the harmful effects of mercury than the adults [38]. Methylmercury may even cross the placenta and cause harm to the developing fetus in pregnant woman [37,38]. Nickel is an immunotoxin and carcinogen [39]. Depending on the dose and length of exposure, nickel can cause allergic reaction and toxicity in the respiratory tract, kidney, lung and immune system [39]. Although vanadium is an element with beneficial effects on humans and holds therapeutic potential in treating diabetes, excessive exposure can cause gastrointestinal problems along with hepato- and nephrotoxicities [40]. Frydas et al. [41] studied gene expression in animal model following exposure to particulate matters (PM) in the air. These particulate matters included various metals and metalloids including V, Zn, As, Pb, Mn, Ni, Cd, Cr and Fe at much lower concentration compared to the concentrations usually exposed through consumption of food, water and air in areas contaminated with heavy metals. Even at the very low concentrations, these metal containing air influenced expression of genes associated with inflammatory response, cell cycle and carcinogenicity [41].

In this study, we used *in silico* tools to analyze 25 global gene expression datasets (comprised of 174 global gene expression profiles in total) of control vs. metal/ metalloid (As, Cd, Cr, Fe, Hg, Ni and V) treated cells of human origin to explore the metabolic pathways that are affected following exposure to arsenic and other heavy metals.

## 2 Methods and materials

### 2.1 Data sources and pre-processing

25 paired global gene expression datasets (control vs. metal treated) from 13 studies in which different human cells (HepG2, MCF7, UROtsa, BEAS-2B, HepaRG, NPrEC and human dermal fibroblasts) were exposed to arsenic, cadmium, chromium, mercury, nickel, iron, and vanadium were retrieved from the National Center for Biotechnology Information (NCBI) Gene Expression Omnibus (GEO) database [42] (Table 1). These studies used different gene expression analysis platforms (Table 1). Other relevant information, such as the form and concentration of the heavy metals used, procedure and duration of exposure to heavy metals, etc are given in Table 1. To avoid the limitations associated with cross-platform data normalization and comparison and reduce ambiguity in the interpretation, we used GEO2R from NCBI GEO to select the top 250 most significantly differentially expressed genes within each paired datasets. Data were filtered at two different levels. First, only those samples that showed comparable median-centered data were included in the analysis (Supplementary Fig. 1). Second, from these samples, only the genes (from the top 250) that had differential gene expression level with false discovery rate (FDR) < 0.05 were selected for further analysis.

**Table 1:** Datasets used in this study.

#	Heavy metal	GEO ID	Platform used	Cell/ Cell line	Used form of heavy metal	Solution	Concentration	Procedure of exposure	Duration of exposure	References
1	As	GSE6907	Affymetrix Human HG-Focus Target Array	HepG2	As^3+^	Arsenic (III) oxide (As_2_O_3_) in Eagle’s Minimal Essential Medium (MEM)	20.0 μM	Cells were cultured in MEM supplemented with fetal bovine serum (FBS), nonessential amino acid, and kanamycin. As_2_O_3_ was added to culture medium as the culture reached ∼70% confluence	6 h	[100]
2	As	GSE8865	Affymetrix Human HG-Focus Target Array	HepG2	As^3+^	Arsenic (III) oxide (As_2_O_3_) in Eagle’s Minimal Essential Medium (MEM)	6.0 μM	Cells were cultured in MEM supplemented with fetal bovine serum (FBS), nonessential amino acid and kanamycin. As_2_O_3_ was added to culture medium as the culture reached ∼70% confluence	48 h	[101]
3	As	GSE48441	Affymetrix Human HG-Focus Target Array	HepG2	As^3+^	Arsenic (III) oxide (As_2_O_3_)	6.0 μM	N/A	48 h	N/A
4	As	GSE48441	Affymetrix Human HG-Focus Target Array	HepG2	As^3+^	Arsenic (III) oxide (As_2_O_3_)	0.5 μM	N/A	48 h	N/A
5	As	GSE136595	Agilent-028004 SurePrint G3 Human GE 8 × 60 K Microarray	MCF7	As^3+^	Sodium arsenite (NaAsO_2_) in Improved Minimal Essential Medium (IMEM)	1.0 μM	Cells were cultured in IMEM supplemented with FBS and glutamine. NaAsO_2_ was added to culture medium	24 h	[102]
6	As	GSE33520	Agilent-014850 Whole Human Genome Microarray 4 × 44 K G4112F	BEAS-2B	As^3+^	Arsenic (III) oxide (As_2_O_3_) spiking solutions in sterile PBS were diluted in fresh DMEM	2.5 μM	Cells were cultured in DMEM supplemented with FBS, L-glutamine and antibiotics (penicillin and streptomycin). As_2_O_3_ was added to culture medium	6 months	[103]
7	As	GSE36684	Affymetrix Human Gene 1.0 ST Array	BEAS-2B	As^3+^	Sodium arsenite (NaAsO_2_) was added to Dulbecco’s Modified Eagle’s Medium (DMEM) from a freshly prepared aqueous filter-sterilized solution	2μM	Cells were cultured in DMEM supplemented with FBS and antibiotics (penicillin and streptomycin). NaAsO_2_ was added to culture medium.	1−2months	[104]
8	As	GSE103873 (GPL16699)	Agilent-039494 SurePrint G3 Human GE v2 8 × 60 K Microarray	HepaRG	As^3+^	Sodium arsenite (NaAsO_2_) in William’s E Medium	1.0 μM	Cells were cultured in William’s E Medium supplemented with growth additives followed by differentiation agents. 3 or 14 days after the initial seeding, NaAsO_2_ was added to the media	>2 weeks	[80]
9	As	GSE103873 (GPL20884)	Agilent-072363 SurePrint G3 Human GE v3 8 × 60 K Microarray	HepaRG	As^3+^	Sodium arsenite (NaAsO_2_) in William’s E Medium	1.0 μM	Cells were cultured in William’s E Medium supplemented with growth additives followed by differentiation agents. 3 or 14 days after the initial seeding, NaAsO_2_ was added to the media.	>2 weeks	[80]
10	Cd	GSE31286	Agilent-014850 Whole Human Genome Microarray 4 × 44 K G4112F	HepG2	Cd^2+^	Cadmium chloride (CdCl_2_) in Opti-MEM medium	10.0 μM	Cells were cultured in Opti-MEM medium supplemented with FBS and antibiotics. CdCl_2_ was added in fresh Opti-MEM 1 day after seeding.	24 h	[105]
11	Cd	GSE6907	Affymetrix Human HG-Focus Target Array	HepG2	Cd^2+^	Cadmium chloride (CdCl_2_) in Eagle’s Minimal Essential Medium (MEM)	2.0 μM	Cells were cultured in MEM and CdCl_2_ was added to culture medium as the culture reached ∼70% confluence	6 h	[100]
12	Cd	GSE9951	Affymetrix Human Genome U133 Plus 2.0 Array	NPrEC	Cd^2+^	Cadmium chloride (CdCl_2_) in Defined Keratinocyte-SFM medium	2.5 μM	Cells were cultured in Defined Keratinocyte-SFM medium supplemented with growth-promoting factors. CdCl_2_ was added in fresh medium 3-days after seeding	16 h	[78]
13	Cd	GSE26828	Affymetrix Human Genome U133 Plus 2.0 Array	UROtsa	Cd^2+^	Cadmium chloride (CdCl_2_) in Dulbecco’s modified Eagle’s medium (DMEM)	1.0 μM	Cells were cultured in DMEM supplemented with FBS. CdCl_2_ was added to fresh culture medium	> 1 month	[106]
14	Cd	GSE31286	Agilent-014850 Whole Human Genome Microarray 4 × 44 K G4112F	HepG2	Cd^2+^	Cadmium chloride (CdCl_2_) in Opti-MEM medium	2.0 μM	Cells were cultured in Opti-MEM medium supplemented with FBS and antibiotics. CdCl_2_ was added in fresh Opti-MEM 1 day after seeding	24 h	[105]
15	Cd	GSE136595	Agilent-028004 SurePrint G3 Human GE 8 × 60 K Microarray	MCF7	Cd^2+^	Cadmium chloride (CdCl_2_) in Improved Minimal Essential Medium (IMEM)	1.0 μM	Cells were cultured in IMEM supplemented with FBS and glutamine. CdCl_2_ was added to culture medium	24 h	[102]
16	Cr	GSE6907	Affymetrix Human HG-Focus Target Array	HepG2	Cr^6+^	Potassium dichromate (K_2_Cr_2_O_7_) in Eagle’s Minimal Essential Medium (MEM)	20 .0μM	Cells were cultured in MEM, and K_2_Cr_2_O_7_ was added to culture medium as the culture reached ∼70% confluence	6 h	[100]
17	Cr	GSE16349	Sentrix Human Ref-8 v2 Expression BeadChip	Dermal fibroblast	Cr^6+^	Potassium dichromate (K_2_Cr_2_O_7_) in culture medium	5.0 μM	K_2_Cr_2_O_7_ was added to culture medium as the culture reached ∼70% confluence	16 h	N/A
18	Cr	GSE24025	Affymetrix Human Gene 1.0 ST Array	BEAS-2B	Cr^6+^	Potassium chromate (K_2_CrO_4_) in culture medium	0.25−0.5 μM	Cells were cultured in DMEM supplemented with FBS and antibiotics (penicillin and streptomycin). K_2_CrO_4_ was added to culture medium	1 month	[107]
19	Cr	GSE36684	Affymetrix Human Gene 1.0 ST Array	BEAS-2B	Cr^6+^	Potassium chromate(K_2_CrO_4_) was added to Dulbecco’s Modified Eagle’s Medium (DMEM) from a freshly prepared aqueous filter-sterilized solution	0.5 μM	Cells were cultured in DMEM supplemented with FBS and antibiotics (penicillin and streptomycin). K_2_CrO_4_ was added to culture medium	1−2 month	[104]
20	Fe	GSE16349	Sentrix Human Ref-8 v2 Expression BeadChip	Dermal fibroblast	Fe^2+^	Iron (II) sulfate (FeSO_4_) in culture medium	40 .0μM	FeSO_4_ was added to culture medium as the culture reached ∼70% confluence	16 h	N/A
21	Hg	GSE6907	Affymetrix Human HG-Focus Target Array	HepG2	Hg^2+^	Mercury (II) chloride (HgCl_2_) in Eagle’s Minimal Essential Medium (MEM)	20.0 μM	Cells were cultured in Eagle’s Minimal Essential Medium (MEM) and HgCl_2_ was added to culture medium as the culture reached ∼70% confluence	6 h	[100]
22	V	GSE36684	Affymetrix Human Gene 1.0 ST Array	BEAS-2B	V^5+^	Sodium metavanadate (NaVO_3_) was added to Dulbecco’s Modified Eagle’s Medium (DMEM) from a freshly prepared aqueous filter-sterilized solution	10.0 μM	Cells were cultured in DMEM supplemented with FBS and antibiotics (penicillin and streptomycin). NaVO_3_ was added to culture medium	1−2 month	[104]
23	Ni	GSE6907	Affymetrix Human HG-Focus Target Array	HepG2	Ni^3+^	Nickel (II) chloride hexahydrate (NiCl_2_ · 6H_2_O)	6.5 mM	Cells were cultured in Eagle’s Minimal Essential Medium (MEM), and As_2_O_3_ was added to culture medium as the culture reached 70% confluence	6 h	[100]
24	Ni	GSE8865	Affymetrix Human HG-Focus Target Array	HepG2	Ni^2+^	Nickel (II) chloride hexahydrate (NiCl_2_·6H_2_O)	150.0 μM	Cells were cultured in MEM supplemented with fetal bovine serum (FBS), nonessential amino acid, fetal bovine serum, and kanamycin. NiCl_2_·6H_2_O was added to culture medium as the culture reached ∼70% confluence	48 h	[101]
25	Ni	GSE36684	Affymetrix Human Gene 1.0 ST Array	BEAS-2B	Ni^2+^	Nickel sulfate (NiSO_4_) was added to Dulbecco’s Modified Eagle’s Medium (DMEM) from a freshly prepared aqueous filter-sterilized solution	250 μM	Cells were cultured in DMEM supplemented with FBS and antibiotics (penicillin and streptomycin). NiSO_4_ was added to culture medium	1−2 month	[104]

### 2.2 Metabolic pathway analysis

The filtered list of genes from each paired dataset was used as an input into the NetworkAnalyst platform [43] and pathways associated with these genes were identified by network enrichment analysis (over representation analysis or ORA) based on the data available in the Kyoto Encyclopedia of Genes and Genomes (KEGG) pathway database [44]. Only the pathways with p < 0.05 were selected for further analysis. Presence or absence of each of these pathways in different datasets was digitized with 1 (for presence) and 0 (for absence). These gene expression derived pathway data were scrutinized by partial least square discriminant analysis (PLS-DA). These datasets were assorted in different combinations to identify the different class specific (depending on the cell line and metals) distinguishing pathways using the PLS-DA Variable Importance in Projection (VIP) scores.

## 3 Results and discussion

### 3.1 Pathways that are affected by particular heavy metals irrespective of the cell lines

**Fig. 1:**
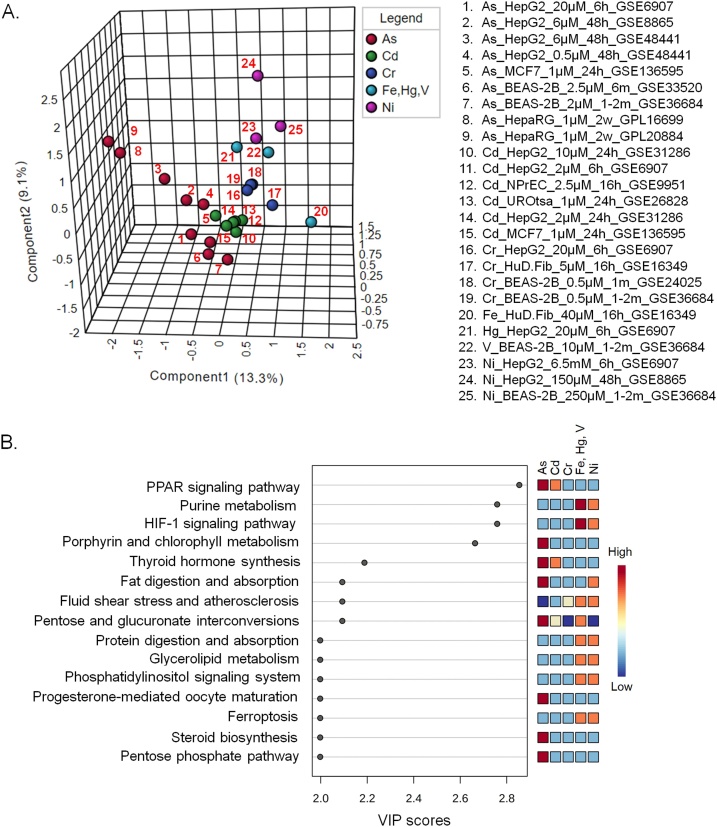
Pathways that are affected by particular heavy metals irrespective of the cell lines. (A) PLS-DA plot of the different cell line derived pathway datasets. B. Heavy metal affected pathways based on the PLS-DA VIP scores.

Irrespective of their tissue of origin, a particular heavy metal treated cell lines tend to cluster together (Fig. 1A). The affected metabolic pathways, however, appear also to be dependent on the heavy metals, which is evident from distribution of different heavy metal treated cells along different axes in the plot (Fig. 1A). Since there was one dataset for each of Fe, Hg and V treatments, these were placed in a single group in the analysis. PLS-DA is a supervised method of multivariate analysis that, in addition to the variances in data, takes into account the class information during the clustering process. Although the class information leads to some level of biasness in the clustering process, this information actually adds benefit in analyses like this where the objective is to identify the features that are responsible for differences between defined groups or clusters.

Fig. 1B shows the pathways that are overly represented in different heavy metal treated cells irrespective of their tissue of origin. Arsenic appears to have the most drastic effect on cellular metabolic pathways. Arsenic and cadmium both affect the expression of genes involved in the PPAR signaling pathway and thyroid hormone biosynthesis. PPARγ plays role in insulin sensitization and is the master regulator of adipogenesis [45,46]. Arsenic also affects the expression of the genes involved in porphyrin metabolism, fat digestion and absorption, progesterone-mediated oocyte maturation, steroid biosynthesis and pentose phosphate pathway (Fig. 1B). Previous studies on arsenic or cadmium mediated toxicities on human and other model organisms also reported several of these affected pathways. For example, multiple studies in the recent years reported diabetogenic effects of arsenic [[47], [48], [49]]. Arsenic-mediated down-regulation of PPARγ in hepatic tissues is involved with the pathogenesis of type 2 diabetes mellitus (T2DM) [50]. Perturbation of PPARγ may also be one of the contributory factors to the carcinogenicity of arsenic [51]. Cadmium inhibits adipocyte differentiation by down-regulating PPARγ [52] and affects placental PPARγ-associated pathway [53]. In addition, cadmium-mediated abnormal adipocyte differentiation, expansion and function, along with hampered insulin signaling may cause insulin resistance, hypertension and cardiovascular diseases [54,55]. High fat diet, an independent factor for development of metabolic syndromes, also increases cadmium accumulation in several organs including testes in cadmium exposed male mice, resulting in increased infertility [56]. Both arsenic and cadmium are involved in PPARγ-dependent apoptosis of rat astrocytes [57]. In addition, both the heavy metal and metalloid can disrupt endocrine regulations [58]. Chronic and combined administration of high fat diet and arsenic reduces production of thyroid hormones in mice [59]. In human, arsenic affects functioning of the thyroid hormone [60,61] as it interferes with thyroid hormone receptor-mediated gene expression regulation [62]. Accumulation of cadmium in the thyroid gland reduces the synthesis of thyroid hormones in both humans and animals [63]. Exposure to cadmium can also result in the development of thyroid cancer [64]. Pentose and glucuronate interconversion pathway is over-represented in arsenic, cadmium, iron, mercury and vanadium treated cells. This pathway is also associated with T2DM [65]. Both arsenic and nickel may affect fat digestion and absorption.

Arsenic exposed cells form a relatively distant cluster from the other heavy metal treated cells (Fig. 1A). This might indicate that a different set of pathways are affected by arsenic. Pathways that are affected by arsenic alone are steroid biosynthesis, pentose phosphate pathway and progesterone-mediated oocyte maturation. A previous study showed that chronic exposure to arsenic can significantly decrease the serum level of progesterone in female mice [66]. Arsenic can become a serious threat to reproductive health of women by acting as an endocrine disruptor and restricting the structure and function of uterus through alterations in gonadotropins and steroid levels at both low and high concentrations [67]. Similarly, arsenic can affect male development and reproduction by altering spermatogenesis, lowering testosterone and gonadotropins and disrupting steroidogenesis [68]. On top of that, arsenic exposure can significantly alter stem cell differentiation pathways including Wnt and Notch signaling, resulting in increased developmental abnormalities, and higher mortality rate of the embryo [69]. Pentose phosphate pathway is a glucose metabolizing pathway, which gives cells the capability to deal with oxidative stress, and thus, may play a role in oxidative stress-induced diabetes [70].

Hg, Ni, Fe, and V affected pathways have many considerable overlaps and include purine metabolism, HIF-1A signaling pathway, fluid shear stress and atherosclerosis, protein digestion and absorption, glycerolipid metabolism, phosphatidylinositol signaling and ferroptosis pathways (Fig. 1B)). The distant position of the only dataset with Fe treatment (#20 in Fig. 1A) from the Ni, Hg and V treated datasets may indicate that this essential nutrient shares fewer common pathways with the other three. Iron plays role in a number of important functions in body such as activation, transport, and storage of molecular oxygen, reduction of ribonucleotides as well as peroxides [71]. But overload with this essential micronutrient for a prolonged period can be toxic to biological systems as it can lead to free radical generation and may mediate damages to heart, liver and exocrine organs including the pancreas [72]. Ferroptosis, a recently described cell death process associated with iron accumulation and lipid peroxidation, which directly or indirectly affect glutathione peroxidase resulting in a decrease in antioxidant capacity and accumulation of lipid reactive oxygen species (ROS) in cells [73], is over-represented in Fe, Hg and V treated cell group. This calls for further investigation to assess whether a similar cellular death process can be induced by other metals like Hg and V.

One distinguishable pathway between Ni and the other (Fe, Hg and V) heavy metal treated cells is the pentose and glucuronate interconversion pathway, which appears to be under-represented in Ni treated cells. This particular pathway is under-represented in the Cr-treated cells as well. Except this one pathway, Cr appears to have the least impact on cellular metabolism. Effects of Fe, Hg, and V on purine metabolism and HIF-1A signaling pathways are more pronounced than those of Ni. In fact, Vanadate- a vanadium oxoanion, is known to induce expression of HIF-1α in a hypoxia-like manner, which in turn leads to progression and metastasis of cancer by increasing expression of vascular endothelial growth factor (VEGF) [74]. Nickel is also known to cause sustained activation of HIF-1α in different human cells [75,76].

As shown in Fig. 1A, there is a tendency of the cell lines treated with a particular heavy metal to cluster closely unless the exposed heavy metal concentration is much different. Also, datasets derived from a particular cell line treated with low concentration of different heavy metals for relatively short period of time tends to appear nearby in the plot (For example, #5 and #15 in Fig. 1A). The two datasets derived from arsenic treated hepatic stem cell line (HepaRG) derived cells cluster together (#8 and #9 in Fig. 1A), but at a distance from the other cell lines. Although HepaRG and HepG2 are human hepatic cell lines, these have different gene expression profiles and consequently show different responses or sensitivity to various toxicants [[77], [78], [79]]. In addition, the tissue origin of HepaRG cell line might have some influence in the effect of heavy metals on metabolism. HepaRG clusters closer to HepG2 than MCF-7 and BEAS-2B (Fig. 1A). Both HepaRG and HepG2 cell lines are used as models to understand hepatotoxicity and xenobiotic metabolism [80,81]. Hence, such clustering pattern might be considered as inherent controls in the study, which is indicative of non-random clustering of the other cell line derived-datasets used.

The distinct cluster of the arsenic treated cells (except #8 and #9 in Fig. 1A) suggests that this metalloid affects similar pathways under different circumstances (chronic vs. acute, and low vs. high load). This appears to be the same for the Cd and Cr treated cells. In addition to the high concentration of iron used, the difference in the origins of the cells might be responsible for the distant placement of Fe treated cells from the rest (Hg and V) in the same group. Differences in the concentration of Ni along with the origin of the cells might be accountable for the relatively loose clustering of Ni-treated cells as well (Fig. 1A).

### 3.2 Dependence of the affected pathways on cell lines, dose and duration of treatment

Datasets used in this study were derived from different cells of human origin. Two of these are human cancer cell lines- HepG2 (from liver cancer cells) [82] and MCF7 (from human breast cancer cells) [83]. HepaRG is a bi-potent hepatic stem cell line [84,85]. BEAS-2B is a human bronchial epithelium derived immortalized, but non-tumorigenic epithelial cell line [86]. NPrEC is a normal prostate epithelial cell line [87] whereas UROtsa is an immortalized normal urothelium cell line [88].

**Fig. 2:**
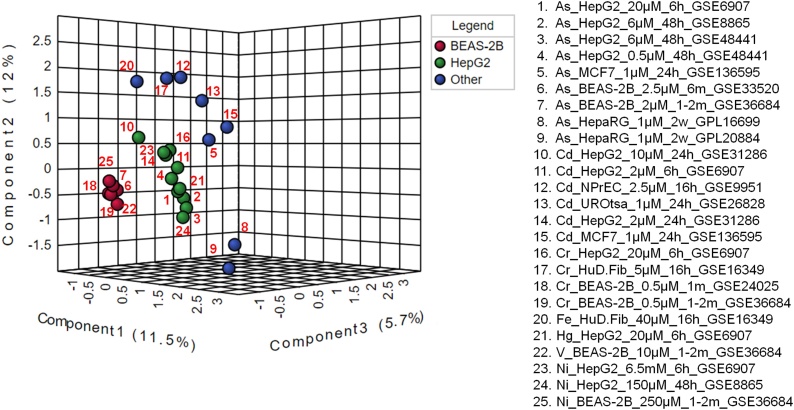
Influence of the origin of cell lines on heavy metal/metalloid mediated effects on pathways. Datasets were analyzed using the PLS-DA method.

**Fig. 3:**
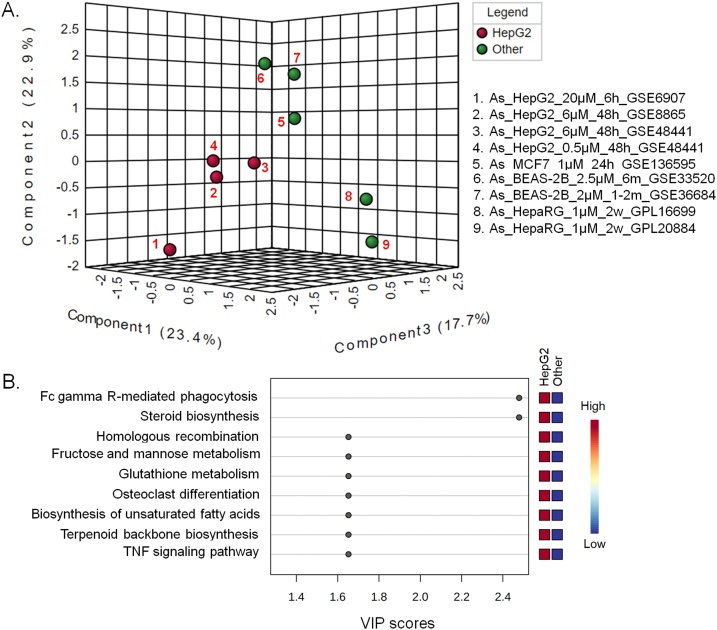
Differentially affected metabolic pathways in HepG2 cells following exposure to arsenic. (A) PLS-DA plot of the different cell line derived pathway datasets. B. Arsenic affected pathways based on the PLS-DA VIP scores.

**Fig. 4:**
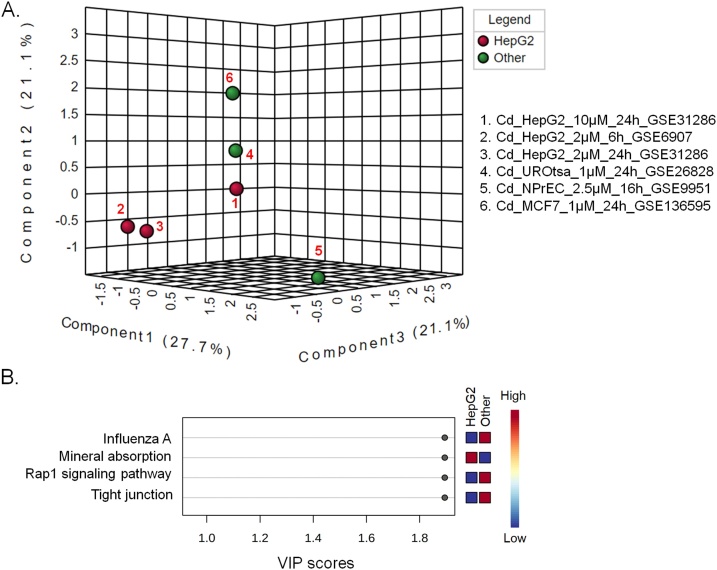
Differentially affected metabolic pathways in HepG2 cells following exposure to cadmium. (A) PLS-DA plot of the different cell line derived pathway datasets. B. Cadmium affected pathways based on the PLS-DA VIP scores.

**Fig. 5:**
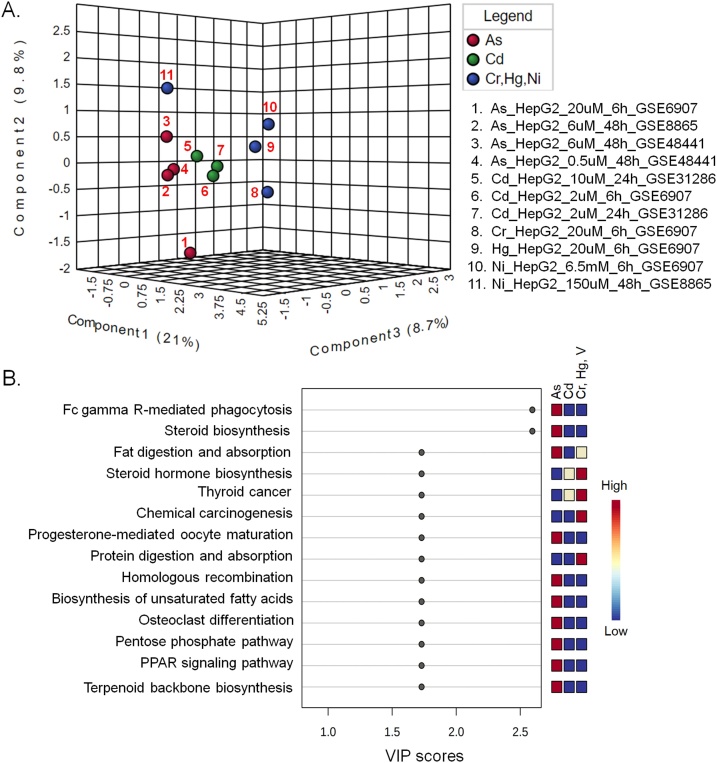
Arsenic and other heavy metals affected metabolic pathways in HepG2 cells. (A) PLS-DA plot of the HepG2 cell line derived pathway datasets. B. Affected pathways based on the PLS-DA VIP scores.

Although different cell lines treated with same heavy metal/metalloid tend to cluster close to one another (Fig. 1A), within-group differences in the positioning of individual datasets is also apparent based on the tissue origin of the studied cell lines (Fig. 2), concentrations of the metals used and duration of exposure (Fig. 3, Fig. 4, Fig. 5). To assess whether the effects of heavy metals is dependent on the tissue of origin, we grouped the datasets based on the cell lines (Fig. 2). Each group, therefore, contained datasets derived from the same cell type despite treatments with different heavy metals/metalloid. Since there were 3 or more datasets for only HepG2 and BEAS-2B cell lines, the rest of the datasets were grouped together. As shown in Fig. 2, datasets of the same cell lines clustered together. Within the “Other” group, the HepaRG datasets grouped distantly from the rest (#8 and #9 in Fig. 2) regardless of the nature of the heavy metal exposure. A study demonstrated that arsenic (in sodium arsenite form) inhibits the differentiation of progenitor like HepaRG into mature hepatocytes and upregulates genes involved in cell growth, proliferation, and survival [89]. In contrast, arsenic down-regulates cell proliferation genes and increase cell death in terminally differentiated hepatocyte-like HepaRG cells [89]. Fig. 1, Fig. 2 reiterate that there may be differences in the affected pathways in different cells of the same tissue origin in response to heavy metal exposure. Likewise, the MCF7 cell derived datasets (#5 and #15 in Fig. 2) clustered closely (Figs. 1A and 2) suggesting the involvement of similar pathways following both arsenic and cadmium treatment. Previous studies also reported that both cadmium and arsenic affect similar processes in MCF7 cell line [[90], [91], [92], [93]]. Similarly, BEAS-2B cell (non-tumorigenic epithelial cell line) derived datasets formed close cluster despite treatment with different heavy metals and metalloid (As, Cr, Ni and V) at varied concentrations (Fig. 2). There appears to be less within-group variation in BEAS-2B group. All these heavy metals and metalloid are directly or indirectly associated with the development of lung cancer [[94], [95], [96], [97]].

### 3.3 Arsenic and cadmium influenced pathways in HepG2 cell lines

Impacts of arsenic on the cellular pathways in HepG2 cell line compared to the other cell lines are shown in Fig. 3. The close clustering of 0.5–6 μM arsenic treated HepG2 cells (#2, #3 and #4 in the Fig. 3A) and their relative distant positions from the 20 μM arsenic treated cells (#1 in Fig. 3) suggest differential impact at high concentration of this metalloid. It can be inferred that this variation in affected pathways might be due to the difference in concentration and duration of exposure. Previous studies also suggested that there may be differences in arsenic-mediated symptoms and complications depending on the duration (acute vs. chronic) and the exposure levels [4,98].

Arsenic has drastically different effects on HepG2 cell line compared to other cell lines (Fig. 3). All the over-represented pathways with VIP score > 1.5 were associated with HepG2 cell line (Fig. 3B). Among the affected pathways Fc gamma receptor (FcγR) mediated phagocytosis and steroid biosynthesis pathways, in particular, distinguish the HepG2 cell line from the other cell lines in the PLS-DA plot (Fig. 3A). Liver is the main organ for the uptake of FcγR mediated antibody-containing immune complexes (ICs) from circulation [99]. Liver is also the major metabolic organ in the body and has crucial role in steroid hormone homeostasis, which is critical in the regulation of various biological pathways, including the reproductive system and responses during stress [100]. Malfunction of these processes may lead to severe liver diseases as well as several endocrine syndromes [100]. Arsenic-induced alterations of steroid metabolism have been reported in mice fetus [101]. Additionally, a previous study reported that chronic low-level arsenic exposure can suppress the expression of Fcγ receptor [102].

The PLS-DA plot along with the overrepresented pathways in cadmium treated cell lines are shown in Fig. 4. Alike arsenic, the concentration of cadmium appears to have an influence on metabolism which is evident from distribution of the HepG2 cells exposed to different concentrations of cadmium. The distant clustering of the other three cadmium-treated cell lines (UROtsa, NPrEC and MCF7) might indicate the influence of tissue origin on the impact of heavy metals.

Compared to arsenic, cadmium has less pronounced effect on metabolic pathways in HepG2 than the other cell lines (Fig. 4). Among the pathways with substantial contribution to observed clustering pattern, only mineral absorption in HepG2 is strongly affected by cadmium (Fig. 4B). Cadmium is in fact known to interfere with calcium metabolism [18].

Cadmium is known to affect tight junctions, influenza A and Rap1 signaling pathways in the other cell lines. Influenza A virus up-regulates the expression of IL-27 in human lung epithelial cells and peripheral blood mononuclear cells through Cyclooxygenase-2 and Protein Kinase A (PKA) signaling [103]. Rap1 participates in the calcium mediated induction of PKA-CREB [103]. Viruses that cause respiratory illnesses, like the influenza A viruses and coronaviruses (specifically, SARS coronavirus), use proteins that target members of the MAGUK family of proteins and disrupt the tight junctions [104]. So, these pathways affected by Cd in the other cell lines might be interconnected.

### 3.4 Effects of different heavy metals on the metabolic pathways in HepG2 cell line

Fig. 5 shows the variation among the pathways in As, Cd, Cr, Hg and Ni mediated toxicities in HepG2 cell line alone. The choice of HepG2 was not arbitrary. HepG2 is the most widely used cell line in metabolic studies and represent the largest number of datasets used in this study. This liver carcinoma cell line is often found to replicate the *in vivo* environment well despite its tumorigenic origin [105]. The pathways affected by arsenic in HepG2 datasets (Fig. 5B) considerably overlap with the pathways affected by arsenic in different cell lines irrespective of tissue origin (Fig. 1A). When the effects of arsenic, cadmium, chromium, mercury and nickel on HepG2 cell line are assessed, more pathways appear to be affected by arsenic (Fig. 5), which matches to the effects observed with the other cell lines as well (Fig. 1). In addition, arsenic appears to affect several other pathways in HepG2 cells including biosynthesis of terpenoids and unsaturated fatty acids, which might be indicative of its tissue of origin. Studies with animal models have shown that arsenic-induced liver injury is accompanied with changes in liver fatty acid profiles [106,107]. Additionally, HepG2 is a model system to study fatty acid synthesis in human liver [108]. Hence, it can be assumed that arsenic can alter fatty acid metabolism in hepatic tissues of human origin.

## 4 Conclusion

This study explored the effects of several commonly encountered heavy metals and metalloid on the metabolic pathways of different cell lines of human origin. The affected metabolic pathways are modulated by not only the heavy metal *per se*, but also the nature and origin of the cells. Therefore, the tissue origin must be considered while assessing the effects of a particular heavy metal/metalloid. Among the heavy metals and metalloid, arsenic appears to have more pleiotropic effects on cellular pathways, including a few known to have association with diabetes. One important limitation of this study is the inability to predict whether the affected pathways were induced or inhibited by the heavy metals. Despite this limitation, this study provides useful information about the pathways that are affected by particular heavy metals and may be directive in further exploration using wet-lab based techniques.

## Statement of ethics

This study neither involved any human nor animal, and hence no ethical approval was required.

## Funding sources

Grant for Advanced Research in Education (GARE) from the Ministry of Education, Bangladesh.

## Author contributions

Design of the work- AAS; Acquisition, analysis, and interpretation of data- KF, SSS, TA, ZH, AFS; Manuscript preparation and reviewing- SSS, KF, TA, AAS

## Declaration of Competing Interest

The authors declare no conflict of interest.

## Figures and Tables

**Figure:**