# Supplementary material for: Effects of arsenic and heavy metals on metabolic pathways in cells of human origin: Similarities and differences
Source: Toxicol Rep. 2021 May 31;8:1109–20. doi: 10.1016/j.toxrep.2021.05.015 (PMC8188178; doi:10.1016/j.toxrep.2021.05.015)
Supplement: Supplementary file 1 [file mmc1.doc]

Supplementary figure 1: Distribution of normalized gene expression data of the selected samples used in this study.
